# Supplementary material for: Combination Effect of Microcystins and Arsenic Exposures on CKD: A Case-Control Study in China
Source: Toxins (Basel). 2023 Feb 10;15(2):144. doi: 10.3390/toxins15020144 (PMC9964595; doi:10.3390/toxins15020144)
Supplement: Supplementary file 1 [file toxins-15-00144-s001.zip › toxins-2082659-supplementary.pdf]

toxins

Article

Combination Effect of Microcystins and Arsenic Exposures on CKD: A Case-Control Study in China

Hong Gao, Na Zhu, Shuxiang Deng, Can Du, Yan Tang, Peng Tang, Shuaishuai Xu, Wenya Liu, Minxue Shen, Xinhua Xiao and Fei Yang

Table S1. Distribution of serum MCs concentration and plasma As concentration according to the case and control groups.

Exposure	Group	Number	Mean	Percentile					$p^†$
				5th	25th	50th	75th	95th	
MCs (μg/L)	Total	270	0.17	0.05	0.12	0.16	0.21	0.32	<0.001
	Case	135	0.19	0.05	0.13	0.16	0.23	0.35	
	Control	135	0.15	0.50	0.10	0.14	0.19	0.29	
As (μg/L)	Total	270	1.51	0.50	0.80	1.12	1.41	3.40	0.003
	Case	135	1.44	0.51	0.88	1.15	1.58	3.55	
	Control	135	1.58	0.49	0.75	1.00	1.31	1.92	

† From Mann-Whitney U test.

Table S2. Distribution of serum MCs concentration and plasma As concentration according to the different eGFR groups.

Exposure	eGFR Groups*	Number	Mean	Percentile					$p^†$
				5th	25th	50th	75th	95th	
MCs (μg/L)	1	7	0.22	0.11	0.14	0.20	0.28	-	<0.001
	2	78	0.21	0.11	0.14	0.17	0.25	0.49	
	3	118	0.14	0.05	0.11	0.14	0.17	0.28	
	4	67	0.17	0.05	0.11	0.17	0.21	0.33	
As (μg/L)	1	7	2.59	0.95	1.23	2.36	3.80	-	<0.001
	2	78	1.59	0.61	1.03	1.28	1.72	4.72	
	3	118	1.64	0.53	0.76	1.02	1.27	1.87	
	4	67	1.09	0.39	0.69	0.93	1.28	2.88	

*The eGFR groups were divided into 1, 2, 3 and 4 according to the range was < 30 mL/min/1.73m^2, 30~59 mL/min/1.73m^2, 60~89 mL/min/1.73m^2 and ≥90 mL/min/1.73m^2, respectively.

Table S3. The probabilities of inclusion derived from the Bayesian Kernel Machine Regression models.

	MCs	As	Cd
Conditional-PIP [a]	0.9991	0.8369	0.8862

[a] This index indicates the posterior probability that a particular exposure was include into the model from the multiple iterations (20000) of the Monte Carlo Markov chain sampler.

Table S4. Sensitivity analysis: Conditional logistic regression analysis and trend test by different outcome diagnostics.

	**Total population AOR (95% CI)**	***p* value[c]**	**Group by eGFR[a] AOR (95% CI)**	***p* value[c]**	**Group by albuminuria[b] AOR (95% CI)**	***p* value[c]**
**MCs[d]**		0.003		0.004		0.34
Q1	reference		reference		reference	
Q2	3.70 (1.38, 9.93)		4.71 (1.36, 16.25)		1.55 (0.72, 3.35)	
Q3	4.20 (1.62, 10.90)		4.96 (1.49, 16.45)		1.78 (0.81, 3.94)	
Q4	4.81 (1.96, 11.81)		6.00 (1.85, 19.48)		1.66 (0.76, 3.62)	
**As[e]**		0.001		0.037		0.18
Q1	reference		reference		reference	
Q2	0.89 (0.37, 2.11)		1.76 (0.74, 4.16)		1.23 (0.55, 2.73)	
Q3	2.51 (1.06, 5.96)		2.04 (0.92, 4.57)		1.46 (0.68, 3.12)	
Q4	3.40 (1.51, 7.65)		2.35 (1.09, 5.07)		1.60 (0.77, 3.34)	

Note: AOR, adjusted odds ratio; CI, confidence interval. The concentrations of MCs/As was divided into the groups of Q1-Q4 according to the quartiles of control group: Q1 (0%-25%), Q2 (25%-50%), Q3 (50%-75%), Q4 (75%-100%). Total population: cases = 135, controls = 135. [a] Case group according to eGFR < 60 mL/min/1.73 m^2: cases = 85, controls = 85. [b] Case group according to albuminuria: cases = 76, controls = 76. [c] *p* for trend was fitted using the median value of MCs or As in each quartile as a continuous variable. [d] Adjusted for hypertension and As concentration. [e] Adjusted for hypertension and MCs concentration.
